# Efficacy and Safety of Injectable Dulaglutide 1.5 mg Among Type 2 Diabetes Patients in Clinics at King Saud Medical City, Riyadh, Saudi Arabia

**DOI:** 10.1007/s44197-024-00207-7

**Published:** 2024-05-16

**Authors:** Mashael Saad Albargawi, Rawan Naser Alharbi, Mohammad Abbas Alajlani, Ibtihal Abdulwarith Abdulaal, Lina Othman Aldakhil

**Affiliations:** 1 Adult Endocrinology Department, King Saud Medical City, Riyadh, Kingdom of Saudi Arabia; 2 Research and Innovation Centre, King Saud Medical City, Riyadh, Kingdom of Saudi Arabia

**Keywords:** T2DM, Dulaglutide, GLP1 agonist, Glycemic control

## Abstract

**Introduction:**

Type 2 diabetes mellitus is the most common type of diabetes, characterized by varying degrees of insulin resistance and diminishing beta-cell function, which increases the risk of macrovascular and microvascular complications. Dulaglutide is a long-acting glucagon-like peptide-1 receptor agonist that is administered once weekly and approved for treating adults with type 2 diabetes mellitus. It can be used as a monotherapy or in addition to oral hypoglycemic or insulin therapy.

**Aim:**

This study aims to provide information contributing to assessing the efficacy and safety of weekly 1.5 mg dulaglutide therapy in Saudi adult patients with type 2 diabetes mellitus.

**Methods:**

A retrospective single-arm cohort study using a purposive sample to recruit type 2 diabetes mellitus patients on dulaglutide from endocrine and diabetic outpatient clinics in King Saud Medical City (*N* = 205). Data were collected from participants’ medical profiles and through the phone using interview questionnaires.

**Results:**

Most participants were female and married; approximately 33% had had diabetes for more than 20 years, 41.4% of the sample had third-class obesity, and more than half had used dulaglutide for the last 1–2 years. With therapy, weight, body mass index, hemoglobin A1c, and fasting blood sugar were significantly improved after 6 and 12 months from baseline. The main side effects reported were nausea (52%) and fatigue (28%).

**Conclusion:**

Dulaglutide is a safe and effective therapy that demonstrated favorable glycemic control and weight reduction in obese type 2 diabetes patients of Saudi origin.

## Introduction

Diabetes mellitus (DM) is a group of metabolic diseases resulting in a high blood sugar level over a prolonged period [1]. It is among the most common global chronic health burdens, affecting many people [2]. Type 2 diabetes mellitus (T2DM) is the most dominant type of diabetes, characterized by varying degrees of insulin resistance and diminishing beta-cell function, which will increase the risk of macrovascular complications, including cardiac disease, cerebrovascular accident, and peripheral arterial disease, as well as microvascular complications of chronic kidney disease, neuropathy, and retinopathy [1–3].

### Background: Diabetes in Saudi Arabia

In 2017, approximately 462 million individuals were diagnosed with T2DM worldwide. This number is expected to reach 642 million by 2040 [1, 4]. Gulf Cooperation Council (GCC) countries have shown growth in diabetes prevalence over the last two decades. According to the World Health Organization, Saudi Arabia has the second-highest prevalence of diabetes in the Middle East and the seventh highest in the world. The last International Diabetes Federation (IDF) report in 2017 showed that the frequency of diabetes in Gulf countries ranged from 8% to 22%. In 2016, the number of Saudi patients diagnosed with diabetes reached 7,000,000. Another 3,000,000 were diagnosed as prediabetic, with the highest death secondary to diabetes compared to other GCC countries [1, 4].

### Treatment Options

Patients with T2DM can be treated by lifestyle modifications and oral antidiabetic drugs (OADs); because T2DM is a progressive disease, maintenance of glycemic targets in some patients with monotherapy is often possible for a few years, after which combination therapy is needed to achieve optimal glycemic control [5]. In addition to insulin, glucagon-like peptide 1 receptor agonists (GLP-1 RAs) are an injectable method for treating T2DM. According to the American Diabetes Association (ADA, 2022), treatment usually starts with lifestyle modification and metformin if there are no contraindications [6]. Additionally, adding another medication, such as GLP-1 RAs, to metformin is recommended in special circumstances, such as in individuals with an established or high risk of cardiovascular or renal complications [7]. Moreover, GLP-1 RAs are a favored injectable complement to insulin in patients with poor glycemic control with OADs and in obese patients [2, 8].

Glucagon-like peptide 1 (GLP-1) is an incretin hormone that increases glucose load-based insulin secretion, reduces glucagon secretion, delays gastric emptying, and decreases appetite, which leads to more glycemic control. GLP-1 RAs have demonstrated glucose-lowering efficacy with a minimal risk of hypoglycemia and marked body weight reduction in several randomized controlled trials (RCTs) [2]. In addition, treatment with GLP-1 RA has significantly reduced cardiovascular disease risk and mortality and improved renal outcomes [2, 5].

Accordingly, the updated ADA 2022 and European Association for the Study of Diabetes (EASD) guidelines recommend adding a GLP-1RA with proven cardiovascular benefit to metformin for patients with high-risk or established atherosclerotic cardiovascular disease (CVD) regardless of whether hemoglobin A1c (HA1c) is at the target level [9].

Dulaglutide is one of these long-acting GLP1 receptor agonists administered as a once-weekly subcutaneous injection approved for the treatment of adults with T2DM in 2014 and can be used as a monotherapy or in addition to an oral hypoglycemic or insulin therapy [5, 8, 10]. It gradually reaches a maximum concentration within 48 h and has a half-life of approximately 5 days. A pharmacokinetic steady state is reached between the second and fourth doses, supporting that no dose adjustment is required based on age, sex, race, body weight, or injection site [11, 12].

### Study Aim and Objectives

The primary objective of this study was to assess the efficacy of weekly 1.5 mg dulaglutide on improving glycemic control and reducing body weight independently after 6 and 12 months from baseline. Other objectives include assessing the possible effect of weekly 1.5 mg dulaglutide on different parameters, such as blood pressure, lipid profile, aspartate-aminotransferase (AST), alanine-aminotransferase (ALT), alkaline phosphatase (ALP), serum creatinine, and estimated glomerular filtration rate (eGFR) levels after 6 and 12 months from the baseline, and exploring the most common dulaglutide side effects reported by T2DM patients, such as nausea, vomiting, diarrhea, abdominal pain, dyspepsia, local injection reaction, and hypoglycemia. We also hope to assess the incidence of serious side effects that can affect patient safety, such as retinopathy, acute pancreatitis, and gallstones.

Because there have been few studies examining this topic in Saudi Arabia, the rationale for conducting this research is to evaluate the efficacy and safety of dulaglutide among Saudi patients with T2DM. It is our hope that doing so will improve the knowledge base about this medication, which other investigators will find useful in future research about dulaglutide in different Saudi regions, which will help improve medical practice and patient safety in Saudi Arabia and globally.

This study aims to provide information contributing to assessing the efficacy and safety of weekly 1.5 mg injections of dulaglutide in Saudi adult patients with T2DM who have been using it for at least 1 year.

## Methods

### Design and Setting

The study design is a retrospective single-arm cohort study. It has been conducted in diabetic clinics at King Saud Medical City (KSMC) in Riyadh, Saudi Arabia. This hospital is considered one of the largest Ministry of Health tertiary hospitals that provide health care to registered populations.

### Target Population

Inclusion criteria included all Saudi adult patients aged more than 18 years with T2DM who had been treated weekly with 1.5 mg dulaglutide for at least 1 year and attended the diabetic clinics at King Saud Medical City. Exclusion criteria included patients with type 1 diabetes, positive glutamic acid decarboxylase (GAD), or islet cell antibodies, and patients who used systematic steroid therapy or chemotherapy while using dulaglutide. Investigators also excluded any patients who had undergone bariatric surgery or used weight-loss medications, patients with eGFR < 30, and any women who became pregnant during their year of using dulaglutide.

### Data Collection Methods

Two methods had been used to collect data.

#### First Interview Questionnaire (Conducted by Phone from May 2023 to August 2023)

To assess dulaglutide safety, the investigators created a new questionnaire to be administered to participants. Data collected after patient consents included personal information, such as age, sex, duration of dulaglutide use, marital status, and education level, along with information about the presence of comorbidities, including hypertension, dyslipidemia (DLP), CVD, and chronic kidney disease (CKD), duration of T2DM, dulaglutide use compliance, common and serious side effects, hypoglycemia events, history of discontinuing use of dulaglutide after 1 year, and if so, the reason for discontinuation.

#### Then, Review of Participant Medical Records (Retrospectively from July 2019 to August 2023)

We retrospectively reviewed the participants’ medical records to evaluate dulaglutide efficacy. We collected data based on patient usage of insulin and oral antihyperglycemic medication and verified the diagnosis of serious side effects by viewing the clinic or emergency department visits and medical progress notes. The body weight, body mass index (BMI), systolic blood pressure (SBP), diastolic blood pressure (DBP), HbA1c level, and other laboratory results, including fasting plasma glucose (FPG), total cholesterol (TC), triglyceride (TG), low-density lipoprotein-cholesterol (LDL), AST, ALT, ALP, serum creatinine, and eGFR, were reported at baseline, 6 months after dulaglutide initiation, and again 12 months after dulaglutide initiation.

### Data Management Plan

In this study, the STATA-13 statistical program was used for data analysis. Categorical descriptive variables such as sociodemographic data (gender, marital status, educational level. etc.), medical profiles, dulaglutide use, and side effects were presented as frequency (percentage) for the difference between weight, BMI, HbA1c, FBS, systolic blood pressure (SBP) and diastolic blood pressure (DBP), Serum Creatinine, GFR, LDL, TG, AST, ALT, and ALP; the repeated one-way ANOVA analysis was used where the mean in time 1, time 2 and time 3 was presented in addition to the mean difference between baseline and 6 months, baseline and 12 months, and between 6 and 12 months.

The data were considered significant at a *p*-value lower than 0.05.

## Results

### Sample Characteristics

In this study, a total of 205 T2DM patients who take a weekly dose of 1.5 mg were included; as Table 1 shows, the sample consisted mainly of females (77.4%) with a mean age of 52 ± 10.88 years, approximately 33% of whom had diabetes for more than 20 years. Moreover, the sample consisted mainly of third-class obese patients (41.44%).

**Table 1:** Sample characteristics and medical history

Gender *n* (%)	
Male	46 (22.55)
Female	158 (77.45)
Age (SD)	52.84 ± 10.88
Marital status *n* (%)	
Single	26 (12.75)
Married	120 (58.82)
Divorce	14 (6.86)
Widow	44 (21.57)
Educational level *n* (%)	
Illiterate	47 (23.04)
Primary	41 (20.10)
Secondary school	41 (20.10)
Intermediate	16 (7.84)
University	42 (20.59)
Higher education	17 (8.33)
Job status *n* (%)	
Employed	60 (29.56)
Unemployed	116 (57.14)
Retired	27 (13.30)
Diabetes duration *n* (%)	
< 5 years	27 (13.30)
5–10 years	48 (23.65)
10–15	34 (16.75)
15–20 years	26 (12.81)
> 20 years	68 (33.50)
BMI *n* (%)	
Normal weight (BMI 18.5 to < 25)	1 (0.55)
Overweight (BMI 25 to < 30)	13 (7.18)
Class I obesity (BMI 30 to < 35)	44 (24.31)
Class II obesity (BMI 35 to < 40)	48 (26.52)
Class III obesity (BMI > 40)	75 (41.44)
Hypertension *n* (%)	
No	77 (37.75)
Yes	127 (62.25)
Dyslipidemia *n* (%)	
No	35 (17.16)
Yes	169 (82.84)
Cardiovascular diseases *n* (%)	
No	160 (78.43)
Yes	44 (21.57)
Chronic kidney disease *n* (%)	
No	197 (96.57)
Yes	7 (3.43)

*BMI* body mass index

### Medical History

As Table 1 presents, more than half of the participants had hypertension (62.2%), and most of them had DLP (approximately 83%); however, a few of the samples had CVD or CKD (21.5% and 3.4%, respectively).

### Dulaglutide Use

Table 2 presents the use of dulaglutide among the patients; according to the table, more than half of the patients use it in 1–2 years period (64.71%); as for the frequency, around 90.69% reported that they always use it, and only 31.86% discontinue using it, out of this about 46% reported that they discount it due their inability to refill the medication 30.7% due side effects, and around 12% due to reaching glycemic target, and around 10% due to other reasons.

**Table 2:** Use of dulaglutide among the participants

Dulaglutide used duration *n* (%)	
1–2 years	132 (64.71)
2–3 years	52 (25.49)
> 3	20 (9.80)
Frequency of using dulaglutide *n* (%)	
Sometimes	2 (0.98)
Often	17 (8.33)
Always	185 (90.69)
Dulaglutide discontinue *n* (%)	
No	139 (68.14)
Yes	65 (31.86)

### Medication Profile

The following table (Table 3) presents the medication used by the participants at the time of the study; as it shows, the most taken medication was metformin, which approximately 94% of the participants took; moreover, long- and short-acting insulin were also commonly used by the participants (51.47% and 38.73%, respectively).

**Table 3:** Patient medication profile

Metformin *n* (%)	
No	13 (6.37)
Yes	191(93.63)
SGLT2 Inhibitors *n* (%)	
No	148 (72.55)
Yes	56 (27.45)
Sulphonylurea *n* (%)	
No	159 (77.94)
Yes	45 (22.06)
Long-acting insulin *n* (%)	
No	99 (48.53)
Yes	105 (51.47)
Short-acting insulin *n* (%)	
No	125 (61.27)
Yes	79 (38.73)
Mixed insulin *n* (%)	
No	184 (90.20)
Yes	20 (9.80)

*SGLT2* sodium-glucose transport protein 2

### Reported Side Effects of Dulaglutide

Table 4 shows that minor and serious side effects have been reported among the participants, as the table shows; aside from nausea, which was the main side effect reported by approximately half of the participants (52%), most of them describe it as rarely experienced (31%) while 15.27% and about 5% reported that they sometimes and often (respectively) get nausea. Fatigue emerged as the second prevalent side effect reported by participants (28%), with the majority describing it as occurring infrequently. Other GI symptoms, such as vomiting, diarrhea, constipation, abdominal pain, and dyspepsia. were also reported rarely, sometimes, and often, and for Constipation, 1.47% always reported. local injection site reaction and low mode were also reported among the sample, with few reporting that they always get these symptoms.

**Table 4:** Reported side effects of dulaglutide

Nausea *n* (%)	
Never	98 (48.28)
Rarely	63 (31.03)
Sometimes	31 (15.27)
Often	10 (4.93)
Always	1 (0.49)
Vomiting *n* (%)	
Never	166 (81.37)
Rarely	28 (13.73)
Sometimes	6 (2.94)
Often	4 (1.96)
Diarrhea *n* (%)	
Never	173 (84.80)
Rarely	17 (8.33)
Sometimes	13 (6.37)
Often	1 (0.49)
Constipation *n* (%)	
Never	172 (84.31)
Rarely	16 (7.84)
Sometimes	9 (4.41)
Often	4 (1.96)
Always	3 (1.47)
Abdominal pain *n* (%)	
Never	176 (86.27)
Rarely	16 (7.84)
Sometimes	11 (5.39)
Often	1 (0.49)
Dyspepsia *n* (%)	
Never	177 (87.19)
Rarely	10 (4.93)
Sometimes	10 (4.93)
Often	5 (2.46)
Always	1 (0.49)
Local injection site reaction *n* (%)	
Never	192 (94.12)
Rarely	8 (3.92)
Sometimes	3 (1.47)
Often	1 (0.49)
Low mode *n* (%)	
Never	155 (75.98)
Rarely	27 (13.24)
Sometimes	13 (6.37)
Often	8 (3.92)
Always	1 (0.49)
Fatigue *n* (%)	
Never	147 (72.06)
Rarely	35 (17.16)
Sometimes	15 (7.35)
Often	5 (2.45)
Always	2 (0.98)
Severity of symptoms *n* (%)	
Mild	86 (63.70)
Moderate	30 (22.22)
Severe	19 (14.07)
Retinopathy *n* (%)	
No	195 (95.59)
Yes	9 (4.41)
Pancreatitis *n* (%)	
No	203 (99.51)
Yes	1 (0.49)
Cholecystitis *n* (%)	
No	202 (99.02)
Yes	2 (0.98)
Gall bladder stone *n* (%)	
No	203 (99.51)
Yes	1 (0.49)
Mild hypoglycemia *n* (%) (glucose level: 70–54 mg/dl)	
Never	151 (74.02)
Rarely	34 (16.67)
Sometimes	15 (7.35)
Often	3 (1.47)
Always	1 (0.49)
Moderate hypoglycemia *n* (%) (glucose level: < 54 mg/dl)	
Never	193 (94.61)
Rarely	9 (4.41)
Sometimes	2 (0.98)
Severe hypoglycemia *n* (%) (with altered mental or physical status and need assistance)	
Never	199 (97.55)
Rarely	4 (1.96)
Sometimes	1 (0.49)

For the more serious symptoms, approximately 4% (*n* = 9) of the participants had retinopathy, while 0.49% (*n* = 1) had pancreatitis, 0.98% reported cholecystitis (*n* = 2), and 0.49% (*n* = 1) reported gall bladder stones.

Furthermore, few participants reported hypoglycemia; 7.35% reported occasional mild hypoglycemia, 0.98% reported moderate hypoglycemia, and 0.49% reported occasional severe hypoglycemia.

### Medical Profile Data Differences Over Time

Table 5 presents the effect of dulaglutide use at baseline, after 6 months, and after 12 months in terms of BMI, HA1c, FBS, SBP, DBP, serum creatinine, eGFR, TC, LDL, TG, AST, ALT, and ALP.

**Table 5:** Medical Profile Data Differences Over Time

	At baseline			After 6 months			After 12 months			*p*-Value
	*N*	Mean	SD	*N*	Mean	SD	*N*	Mean	SD	
Weight (kg)	184	100.94	± 20.44	166	96.14	± 20.67	191	93.29	± 20.16	< 0.001
BMI (kg/m^2^)	184	39.38	± 7.90	164	37.59	± 7.80	186	36.53	± 7.87	<0.001
HbA1c (%)	200	9.18	± 1.97	181	8.03	± 1.72	188	7.75	± 1.72	<0.001
FBS (mg/dL)	188	10.60	± 4.29	178	9.15	± 4.09	186	8.52	± 3.58	< 0.001
SBP (mmHg)	158	137.16	± 17.67	147	136.73	± 19.87	161	134.32	± 18.90	0.16
DBP (mmHg)	158	78.67	± 11.00	147	78.13	± 12.25	160	77.90	± 11.50	0.64
Serum creatinine (μmol/L)										
Male	42	81.44	± 24.10	40	80.84	± 24.40	41	84.49	± 23.75	0.51
Female	152	57.68	± 15.89	138	61.78	± 19.16	147	60.88	± 16.12	< 0.001
eGFR (mL/min/1.73 m^2^)	193	101.01	± 18.46	178	98.75	± 20.48	188	98.24	± 20.07	0.00
TC (mmol/L)	186	4.55	± 1.09	174	4.37	± 1.07	177	4.37	± 1.07	0.02
LDL (mmol/L)	176	2.64	± 1.12	157	2.38	± 0.92	161	2.37	± 0.93	0.01
TG (mmol/L)	185	1.77	± 0.89	168	1.74	± 0.92	173	1.74	± 0.94	0.43
AST (IU/L)	182	19.76	± 10.96	157	18.13	± 8.30	167	18.61	± 6.96	0.33
ALT (IU/L)	184	23.59	± 12.18	157	21.42	± 13.05	168	21.64	± 13.91	0.13
ALP (IU/L)	187	95.16	± 33.34	165	87.40	± 31.16	170	87.73	± 26.99	0.00

*BMI* body mass index; *Hba1c* hemoglobin A1C; *FBS* fasting blood sugar; *SBP* systolic blood pressure; *DBP* diastolic blood pressure; *eGFR* estimated glomerular filtration rate; *TC* total cholesterol; *LDL* low-density lipoproteins; *TG* triglycerides; *AST* aspartate aminotransferase; *ALT* alanine aminotransferase; *ALP* alkaline phosphatase

As the table shows, there were significant differences in weight, BMI, HA1c, FBS, serum creatinine in females, eGFR, total cholesterol, LDL, and ALP.

At baseline, the mean weight among the participants was 100.94 kg (± 20.44); after 6 months, the average weight was 96.137 kg (± 20.67); and after 12 months, the average weight fell to 93.292 kg (± 20.16). This difference is statistically significant (*p*-value < 0.001).

BMI showed a significant difference (*p*-value < 0.001) over time, from the baseline average BMI of 39.38 (± 7.90) kg/m^2^ to an average BMI of 37.59 (± 7.8) kg/m^2^ at 6 months, falling to 36.52 (± 7.87) kg/m^2^ at 12 months.

FBS also shows a significant difference (*p*-value < 0.001) over time. At baseline, the mean was 10.60 mg/dL (± 4.29); after 6 months, it was 9.146 mg/dL (± 4.097) mg/dL; and at 12 months, it was 8.51 (± 3.58) mg/dL.

Serum creatinine for females also showed a significant difference (*p*-value < 0.001). There is a steady increase from baseline to 6 months, with the average level rising from 57.6 (± 15.8) μmol/L at baseline to 61.78 (± 19.16) μmol/L at 6 months. However, this level decreased, on average, between 6 and 12 months to an average mean of 60.8 (± 16.12) μmol/L.

The estimated GFR was significantly (*p* < 0.001) lower after 6 months than at baseline (98.75 ± 20.4 mL/min/1.73 m^2^ and 101 ± 18.46 mL/min/1.73 m^2^, respectively). The same goes for TC, which fell from an average total cholesterol of 4.550 (± 1.09) mmol/L at baseline to 4.366 (± 1.071) mmol/L at 6 months.

The difference in LDL was statistically significant (*p*-value < 0.05) but minor overall, with 2.64 (± 1.12) mmol/L at baseline dropping slightly to 2.383 (± 0.924) mmol/L after 6 months and 2.37 (± 0.935) mmol/L at 12 months.

Finally, for the ALP, it was found to be a statistically significant difference with a noticeable change after 6 months of 87.40 (± 31.16) IU/L.

### Differences Within the Group Over Time

Table 6 shows the differences within the group in terms of weight, BMI, HA1c, FBS, eGFR, TC, LDL, and ALT between baseline and after 6 months, between baseline and after 12 months, and between 6 and 12 months. We see a significant difference between weights; it appears that the highest difference was found to be between baseline and after 12 months with a mean difference of 8 kg [95% CI: (6.57, 9.59)] with *p*-value < 0.001; the difference in mean weight is smaller between 6 and 12 months, with a mean difference of 2.5 kg [95% CI: (1.67, 3.34)].

**Table 6:** Mean differences within the group over time

Weight (kg)		Mean difference	95% CI		*p*-Value
At baseline	At 6 months	5.57	4.40	6.75	< 0.001
At baseline	At 12 months	8.08	6.57	9.59	< 0.001
At 6 months	At 12 months	2.51	1.67	3.34	< 0.001
BMI (kg/m^2^)					
At baseline	At 6 months	2.19	1.73	2.65	< 0.001
At baseline	At 12 months	3.16	2.55	3.76	< 0.001
At 6 months	At 12 months	0.96	0.61	1.32	< 0.001
HbA1c (%)					
At baseline	At 6 months	1.12	0.86	1.39	< 0.001
At baseline	At 12 months	1.43	1.16	1.70	< 0.001
At 6 months	At 12 months	0.31	0.10	0.52	0.01
FBS (mg/dL)					
At baseline	At 6 months	1.18	0.40	1.96	0.01
At baseline	At 12 months	1.83	1.06	2.59	< 0.001
At 6 months	At 12 months	0.65	−0.01	1.30	0.15
eGFR (mL/min/1.73 m^2^)					
At baseline	At 6 months	2.90	1.20	4.59	0.00
At baseline	At 12 months	3.17	1.08	5.25	0.01
At 6 months	At 12 months	0.27	−1.80	2.35	1.00
TC (mmol/L)					
At baseline	At 6 months	0.25	0.04	0.45	0.05
At baseline	At 12 months	0.26	0.05	0.47	0.05
At 6 months	At 12 months	0.01	−0.17	0.20	1.00
LDL (mmol/L)					
At baseline	At 6 months	0.32	0.07	0.58	0.04
At baseline	At 12 months	0.33	0.08	0.58	0.04
At 6 months	At 12 months	0.01	−0.16	0.17	1.00
ALP (IU/L)					
At baseline	At 6 months	7.25	2.03	12.47	0.02
At baseline	At 12 months	5.53	1.36	9.69	0.03
At 6 months	At 12 months	−1.72	−5.42	1.98	1.00

*BMI* body mass index; *Hba1c* Hemoglobin A1C; *FBS* fasting blood sugar; *eGFR* estimated glomerular filtration rate; *TC* total cholesterol; *LDL* low-density lipoproteins; *ALP* alkaline phosphatase

BMI showed a statistically significant difference at all three points (*p*-value < 0.05). The mean difference was largest between baseline and 6 months and between baseline and 12 months (mean difference: 2.19 and 3.16 kg/m^2^, respectively).

The same goes for HA1c; the difference is more prevalent after the first 6 months, where it is 1.12 (95% CI: 0.86, 1.39) lower. However, the level stabilized between 6 and 12 months, with a mean difference of just 0.31 (95% CI: 0.1, 0.52), although it was significant within the group (*p*-value < 0.05).

The difference in FBS level was significantly lower between baseline and 6 months and between baseline and 12 months only, with *p*-values < 0.05 (1.18 and 1.83 mg/dL, respectively).

Moreover, the mean difference in eGFR was significantly increased only between baseline and 6 months [mean difference: 2.9 mL/min/1.73 m^2^ (95% CI:1.2, 4.5)] and between baseline and 12 months [mean difference: 3.17 mL/min/1.73 m^2^ (95% CI: 1.08, 5.25)], with a *p*-value < 0.05.

TC only showed a significant change (*p*-value < 0.05) when viewed in the long term, with a mean difference of 0.26 mmol/L and 95% CI (0.05, 0.47) between baseline and 12 months.

The same goes for LDL and ALT, as the mean difference is significant between baseline and 6 months and between baseline and 12 months only.

## Discussion

Dulaglutide, an approved long-acting GLP-1 RAs administered weekly, is indicated for treating adults with type 2 diabetes mellitus. Previous trials substantiated its effectiveness in glycemic control and weight reduction in obese patients [2]. Our study affirmed these findings, demonstrating a notable decrease in HA1c, FBS, and weight among patients using dulaglutide for a minimum of one year.

Dulaglutide has been assessed in several phase III trials, known as the global AWARD (Assessment of Weekly Administration of LY2189265 [dulaglutide] in diabetes) studies, showing that dulaglutide is generally an effective medication in maintaining glycemic control as monotherapy or if added to insulin or oral together with sustained weight loss and a low rate of hypoglycemia [5, 9, 13].

The Award 1–5 program, including phase 3 trial studies for dulaglutide, demonstrated mean weight loss ranging between 0.4 and 2.9 kg with a dulaglutide dose of 1.5 mg weekly for 26–52 weeks, either as monotherapy or in comparison with sitagliptin, exenatide or insulin glargine [14, 15]. Similar findings have been shown from several real-world studies regarding the efficacy of dulaglutide in lowering glucose with body weight reduction in patients with T2DM, including high-risk patients such as the elderly, patients with stage 3 or 4 chronic kidney disease, and patients with cardiovascular disease [10]; the most recent study was conducted in 2021 for Korean patients with T2DM and evaluated the glycemic efficacy of dulaglutide as an add-on to oral antidiabetic drugs (OADs) and basal insulin for 6 months [2, 16]. Our study demonstrates similar results in HbA1c reduction and slightly higher weight reduction, with a mean reduction of HbA1c by 1.12 after 6 months and 1.43% after 12 months and a body weight reduction of 5.57 kg after 6 months and approximately 8 kg after 12 months.

The Researching Cardiovascular Events with a Weekly Incretin in Diabetes (REWIND) trial assessed cardiovascular outcomes in diabetic patients on 1.5 mg dulaglutide and showed cardio and renal protection in patients with T2DM who are at high risk of cardiovascular events [13, 17]. As per our study, we did not assess the effect of dulaglutide on cardiovascular and renal protection; however, we observed a significant decrease in estimated glomerular filtration rate (eGFR) after 6 months compared to baseline (98.7 ± 20.4 and 101 ± 18.4 mL/min/1.7 m^2^, respectively), with a *p*-value less than 0.001. These findings may be explained by the chronicity of T2DM and its micro- and macrovascular complications. On the other hand, we also reported significant reductions in SBP, TC, and LDL levels in our study after 6 and 12 months. These results support similar findings reported in a post hoc analysis of the AWARD-11 study, conducted for participants who received once-weekly dulaglutide 1.5, 3.0, or 4.5 mg for 52 weeks, and showed that dulaglutide resulted in dose-related decreases in TC (≤ 6.0%) and TG (≤ 21.5%), with little change in LDL. SBP and DBP decreased up to 5.6 and 1.6 mmHg, respectively. These reductions have a positive impact on preventing cardiovascular disease [18]. Furthermore, extensive longitudinal studies must be conducted to assess the cardiovascular and renal benefits among Saudi patients.

Early mild gastrointestinal symptoms, including nausea, diarrhea, and vomiting, were the most reported adverse effects of dulaglutide in most AWARD studies, regardless of the injection doses, with a peak during the first 2 weeks of starting medication and then a rapid decline over the next 4 weeks. Other GI-related events, such as constipation, dyspepsia, and abdominal pain, were also reported [9]. However, the incidence of drug discontinuation due to severe GI events was rare, reaching 1.5% in participants taking 1.5 mg compared to 3.9% in participants taking 4.5 mg, as in the AWARD 2 study [3]. This was confirmed in our study, which showed nausea as the main side effect reported among participants, followed by other GI symptoms; approximately 31.8% of participants discontinued using dulaglutide after 12 months; of these, 30.7% (*n* = 18) cited side effects as their reason for discontinuing.

Six cases of pancreatitis were confirmed in the Award 2 study, and similar proportions of participants reported adverse events in the gallbladder. However, there were no reports of pancreatic cancer, C-cell hyperplasia, or medullary thyroid carcinoma during the study period in AWARD 2 and 3 [3, 16]. Participants reported a pancreatic carcinoma in the dulaglutide 1.5 mg treatment group in the AWARD 1 study, and one participant reported medullary thyroid carcinoma (MTC) in the dulaglutide 2.0 mg treatment group after approximately 6 months of starting medication in the AWARD 5 study [19]. In our study, one participant only experienced pancreatitis, one experienced gallbladder stones, and two reported cholecystitis; it was not definitively established whether those conditions were related to dulaglutide use. In general, these serious side effects are infrequent.

We found that nine participants experienced retinopathy after starting dulaglutide. The duration of dulaglutide use before retinopathy diagnosis is not precisely known because many diabetic patients are not routinely followed by ophthalmology, and some do not have a baseline retinal examination before starting dulaglutide. By reviewing previous trials, we found that a temporary worsening of diabetic retinopathy was reported in the REWIND study (8.5%) in the participants on dulaglutide, and it was reported in participants with a baseline history of diabetic retinopathy. There is no evidence that the GLP-1RA class has any direct harmful effect on the development or progression of diabetic retinopathy [9].

Multiple studies have also reported injection site reactions with mild symptoms, including pain, pruritus, and rash at the injection site [15]. Among our study population, only 12 participants reported some form of injection site reactions, which were mild and tolerable.

The incidence of documented severe hypoglycemia (i.e., plasma glucose of 54 mg/dL [3.0 mmol/L]) was very low in the comparison studies, reaching 1.3% of events per year in participants on dulaglutide 1.5 mg in the AWARD 2 study; no patient discontinued from the study due to hypoglycemia [9, 20]. In general, hypoglycemia was rarely reported in our findings, and most of those instances (7.35% of the total study population) were mild hypoglycemia, and only one participant reported severe hypoglycemia.

For liver enzymes, we reported a significant reduction only in ALP levels after 6 and 12 months from baseline, with means of 7.25 and 5.53 IU/L, respectively; these reductions were also reported in one meta-analysis study involving 12 studies with GLP-1RA arms and 677 subjects that showed that treatment with GLP-1RA reduced ALT, gamma-glutamyl transferase (GGT), and ALP with no change in AST level, leading to the conclusion that GLP-1RA treatment significantly reduces liver enzymes in patients with NAFLD [21].

There are inherent limitations in our study, as it is a single-arm study without either structured randomization or control groups, which can reduce the validity and significance of the study results. The study duration was short, which limits the strength of the outcome when assessing long-term safety and efficacy. Additionally, a retrospective cohort design can be associated with bias and confounding factors, which must be considered when interpreting findings.

However, despite the study’s limitations in its scope and duration, it achieved its primary objective, which was to assess the efficacy of once-weekly 1.5 mg dulaglutide therapy in improving glycemic control and weight loss in Saudi patients. It also provides practical and significant information to healthcare professionals in Saudi Arabia.

## Conclusion

Our study has demonstrated that dulaglutide is a safe and effective treatment for improving glycemic control and reducing weight in people with T2DM and obesity of Saudi origin. Most side effects are mild and tolerable and mainly consist of GI symptoms and fatigue. The risk of severe side effects and hypoglycemia is rare, as reported both in our study and in previous real-world trials.

Further large-scale studies—preferably encompassing multiple centers from different cities in Saudi Arabia with larger study cohorts and long durations and assessing cardiovascular effects and cost benefits—are needed to support the utility of dulaglutide in the Saudi population.

## Data Availability

Original data generated and analyzed during this study are included in this published article.

## Abbreviations

ALT: Alanine aminotransferase
ALP: Alkaline phosphatase
AST: Aspartate aminotransferase
AWARD: Assessment of weekly administration of LY2189265 [dulaglutide] in diabetes
BMI: Body mass index
CVD: Cardiovascular disease
CKD: Chronic kidney disease
DM: Diabetic mellitus
DBP: Diastolic blood pressure
DLP: Dyslipidemia
eGFR: Estimated glomerular filtration rate
EASD: European Association for the Study of Diabetes
FBS: Fasting blood sugar
GGT: Gamma-glutamyl transferase
GI: Gastrointestinal
GLP-1 RAs: Glucagon-like peptide 1 receptor agonists
GLP-1: Glucagon-like peptide 1
GAD: Glutamic acid decarboxylase
GCC: Gulf Cooperation Council
HA1c: Hemoglobin A1c
ICA: Islet cell antibodies
KSMC: King Saud Medical City
LDL: Low-density lipoprotein-cholesterol
OADs: Oral antidiabetic drugs
RCTs.: Randomized controlled trials
REWIND: Researching cardiovascular events with a weekly incretin in diabetes
SGLT2: Sodium-glucose transport protein 2
SBP: Systolic blood pressure
ADA: The American Diabetes Association
TC: Total cholesterol
TG: Triglyceride
T2DM: Type 2 diabetes mellitus
